# Developing a tactical irrigation and nitrogen fertilizer management strategy for winter wheat through drip irrigation

**DOI:** 10.3389/fpls.2023.1231294

**Published:** 2023-08-10

**Authors:** Muhammad Zain, Zhuanyun Si, Haijiao Ma, Minghan Cheng, Adam Khan, Faisal Mehmood, Aiwang Duan, Chengming Sun

**Affiliations:** ^1^ Key Laboratory of Crop Genetics and Physiology of Jiangsu Province, Key Laboratory of Crop Cultivation and Physiology of Jiangsu Province, College of Agriculture, Yangzhou University, Yangzhou, China; ^2^ Key Laboratory of Crop Water Use and Regulation, Farmland Irrigation Research Institute, Chinese Academy of Agricultural Sciences, Ministry of Agriculture and Rural Affairs, Xinxiang, Henan, China; ^3^ Department of Botany, University of Lakki Marwat, Lakki Marwat, Pakistan; ^4^ Department of Land and Water Management, Faculty of Agricultural Engineering, Sindh Agricultural University, Tandojam, Pakistan

**Keywords:** drip irrigation, irrigation scheduling, lateral spacing, nitrogen fertilization scheme, winter wheat

## Abstract

**Introduction:**

Agricultural activities in the North China Plain are often challenged by inadequate irrigation and nutrient supply. Inadequate and improper resource utilization may impose negative impacts on agricultural sustainability. To counteract the negative impacts, a deeper understanding of the different resource management strategies is an essential prerequisite to assess the resource saving potential of crops.

**Methods:**

We explored plausible adaptation strategies including drip irrigation lateral spacings of 40 and 80 cm (hereafter referred to as LS_40_ and LS_80_, respectively), irrigating winter wheat after soil water consumption of 20 and 35 mm (hereafter represented as IS_20_ and IS_35_, respectively), and nitrogen fertilization scheme of a) applying 50% nitrogen as a basal dose and 50% as a top-dressing dose (NS_50:50_), b) 25% nitrogen as a basal dose and 75% as a topdressing dose (NS_25:75_), and c) no nitrogen application as a basal dose and 100% application as a top-dressing dose (NS_0:100_).

**Results and discussion:**

The consecutive 2 years (2017–2018 and 2018–2019) of field study results show that growing winter wheat under LS_40_ enhanced the water use efficiency (WUE), grain yield, 1,000-grain weight, and number of grains per spike by 15.04%, 6.95%, 5.67%, and 21.59% during the 2017–2018 season, respectively. Additionally, the corresponding values during the 2018–2019 season were 12.70%, 7.17%, 2.66%, and 19.25%, respectively. Irrigation scheduling of IS_35_ treatment improved all the growth-related and yield parameters of winter wheat. Further, treating 25% nitrogen as a basal dose and application of 75% as a top-dressing dose positively influenced the winter wheat yield. While NS_0:100_ increased the plant height, leaf area index (LAI), and aboveground biomass as compared to the other application strategies, but high nitrogen was observed in deeper soil layers. Regarding soil environment, the lowest soil moisture and nitrate nitrogen contents were observed in LS_80_ during both growing seasons. Overall, coupling the IS_35_ with NS_25:75_ under 40-cm lateral spacing is a suitable choice for sustainable winter wheat production in theNorth China Plain. The results of our study may be helpful in advancing the knowledge of the farmer community for winter wheat production. The findings can also aid in advancing new insights among scientists working on soil water and nitrogen distribution in drip irrigation for better productivity.

## 1 Introduction

The burgeoning population demand for more supply of staple food, energy, and water is a serious challenge. This challenge is more chronic in regions where water resources are less and food demand is high. Wheat (*Triticum aestivum* L.) is a chief food source having a share of 20% in global calorific consumption by the world’s population (Liu et al., 2020a; Liu et al., 2020b). According to The Food and Agriculture Organization of the United Nations (FAO) (2020), China is a leading wheat producer in the world with 134.2 million tons of production on an area of 23.3 million hectares in 2020. Additionally, the North China Plain (NCP) produces approximately 50%–61% of China’s total wheat production (Wang et al., 2012). Sustainable water management is a big challenge in NCP, especially where 70% of freshwater is being exploited for agricultural activities and the groundwater table is dropping at a rate of approximately 1 m/year (Du et al., 2015; Yang et al., 2017). However, water scarcity due to low precipitation and drought especially during the winter wheat growth period is a major reason to limit the wheat yield in NCP (Si et al., 2020). Water deficiency negatively affects crop productivity, as drought condition leads to many physiological and biochemical reactions in plants (Jiang et al., 2020; Sidhu et al., 2021). Therefore, water-saving practices that give sustainable crop productivity and improve water use efficiency (WUE) should be developed.

Drip irrigation systems that have high performance are encouraged to tackle water scarcity challenges and for high water productivity as opposed to conventional irrigation methods. Previously, drip irrigation has been applied to low-density crops like cotton and corn and in fruit production. Currently, the application of drip irrigation in cereals was gradually adopted to resolve the water shortage issues in arid and semi-arid climates (Dar et al., 2017; Mostafa et al., 2018). Many scientists have reported drip irrigation application in wheat crops and found that drip irrigation saves more water compared to conventional irrigation methods. For example, Li et al. (2018) compared the traditional flood irrigation method with drip irrigation and found that wheat plants under drip irrigation have high root density, which improves the water and nutrient absorption in deep soil layers. Increasing irrigation frequency under drip irrigation sustains high moisture, especially in upper soil layers, and enhances crop productivity (Fang et al., 2018). Drip irrigation is conducive to saving irrigation water and in the improvement of dry matter accumulation, crop photosynthesis, and ultimately crop productivity (Cao et al., 2021; Li et al., 2021; Yan et al., 2022). In a comparison of drip irrigation with border irrigation by Yang et al. (2020), it was noticed that drip irrigation decreased soil evaporation, transpiration, and wheat evapotranspiration by 18.5, 22 mm, and 3.5 per year, respectively. Drip irrigation improves the winter wheat yield by 5%–13% and significantly saves irrigation water of approximately 45.9–114.8 mm, which ultimately improves the WUE as compared to the level-basin irrigation method (Wang et al., 2013). Wang et al. (2013) further added that more water productivity can be achieved through drip irrigation by maintaining the soil moisture up to 50%–60% field capacity.

Uniform distribution of water and nutrients around the drip tubes must be followed in order to gain high crop productivity and resource use efficiency. The consistent distribution of moisture under a drip irrigation system is remarkably dependent on the strip width or lateral spacings between the drip irrigation pipes and also on other irrigation strategies such as intervals between irrigation and irrigation levels (Payero et al., 2008; Wang et al., 2013). To date, there are few studies on drip irrigation systems in winter wheat. Thus, more research is required to assess the performance of drip irrigation systems for sustainable agricultural development in NCP.

Fertilizer application, especially nitrogen supply, also directly or indirectly exerts an impact on normal crop growth and productivity (Jia et al., 2014; Wang et al., 2021). Usually, farmers apply nitrogen fertilizer according to their own know-how without worrying about environmental penalties. Lassaletta et al. (2014) stated that immoderate nitrogen application leads to a loss of nitrogen by >50%, which causes environmental contamination. Ju et al. (2009) also stated that denitrification, nitrate nitrogen leaching, and ammonia volatilization cause 15%–45% of the nutrient loss in NCP, which might lead to non-point source groundwater contamination. Inappropriate nitrogen fertilization not only disturbs the environmental compartments but also becomes a factor of high economic inputs. The strategic application of nitrogen to wheat crops may extend the grain-filling stage and enhance the photosynthetic capacity, which results in better productivity (Zhang et al., 2020). Blandino et al. (2015) reported that top dressing of nitrogen can improve wheat productivity as compared to the basal application, while they further added that basal nitrogen application results in nitrogen loss via volatilization. Further, Chen et al. (2018) found that the application of nitrogen in 4:4:2 ratios at the sowing, jointing, and anthesis stages remarkably improved the yield as compared to the 4:6 and 6:4 application ratios. However, there is no clear information about nitrogen application for winter wheat specifically in NCP.

Many studies reported that water and nitrogen management approaches show interactive effects on crop growth and productivity, but these studies were conducted under conventional irrigation systems (Fang et al., 2013; Hamzei, 2018). Fertigation is an advanced technology that saves resources and provides more yield by delivering water and nutrients directly to the root zone (Wolff et al., 2017) and reducing leaching losses (Gärdenäs et al., 2005). Further, nitrogen distribution in the soil varies in drip irrigation as opposed to conventional flooding methods, and nitrogen distribution essentially depends on water movement and application methods (Gasser et al., 2002). Therefore, it is crucial to find out the best irrigation scheduling practice in integration with the nitrogen application scheme on wheat productivity in NCP.

Although extensive work on irrigation scheduling has been performed in this region for better winter wheat production (Jha et al., 2019; Mehmood et al., 2019; Si et al., 2020), to date, efforts have not been made to determine the combined effects of nitrogen application schemes and irrigation scheduling on wheat production under different drip irrigation lateral spacings. Thus, the current field experiment aimed to i) investigate the suitable nitrogen application strategy and irrigation scheduling for better winter wheat productivity, ii) quantify the distribution of soil moisture and nitrogen under different lateral spacings at various irrigation and nitrogen treatments, and iii) reveal the consequences of different irrigation scheduling and nitrogen treatments on growth and yield of wheat and water use efficiency under various lateral spacings.

## 2 Materials and methods

### 2.1 Experimental site description

The experiment was conducted in 2017–2018 and 2018–2019 growing seasons of winter wheat at the Experimental Station of Farmland Irrigation Research Institute, Chinese Academy of Agricultural Sciences (CAAS). The experimental site is located in Qiliying (35°08′N, 113°45′E; altitude 81 m) in Xinxiang City of Henan province, PR China. The physicochemical properties of the experimental site are given in **Table 1**. Soil texture is sandy loam and alkaline (8.5 pH) with 257.6 μs/cm electrical conductivity and is low in organic matter (1.10%). The available nitrogen, phosphorous, and potassium contents in the soil profile (average of 1-m soil depth) are 40.20, 11.90, and 100.51 mg/kg, respectively.

**Table 1:** Particle size distribution, bulk density, permanent wilting point, and field capacity of the experimental site at 1-m depth.

Layer (m)	Particle size distribution			Soil texture	BD (g/cm^-3^)	PWP (cm^3^/cm^3^)	FC (cm^3^/cm^3^)
	Sand (%)	Silt (%)	Clay (%)				
0–0.2	53.06	43.14	3.80	Sandy loam	1.56	0.163	0.341
0.2–0.4	47.96	45.43	6.61	Loam	1.58	0.157	0.308
0.4–0.6	45.61	48.33	6.06	Sandy loam	1.54	0.181	0.327
0.6–0.8	47.96	47.49	4.55	Sandy loam	1.42	0.181	0.283
0.8–1.0	81.48	16.95	1.57	Loamy sand	1.45	0.173	0.294
Average	55.21	40.27	4.52	Sandy loam	1.51	0.171	0.311

BD, bulk density; PWP, permanent wilting point; FC, field capacity.

**Figure 1:**
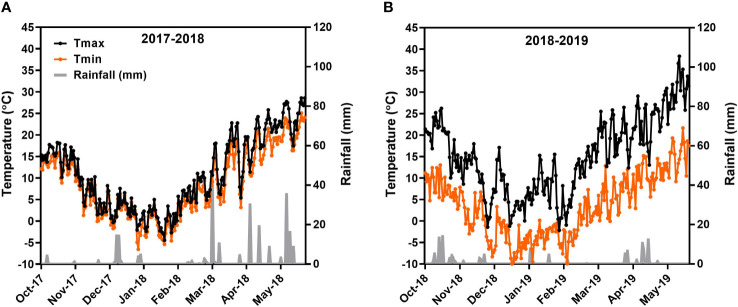
Weather situations during the course of field experiments. Part **(A)** represents 2017–2018 growing season, and part **(B)** represents 2018–2019 growing season.

The weather data including air temperature (both maximum and minimum) and precipitation were collected on a daily basis from a meteorological station located at the experimental site. Generally, the rainfall at the experimental site during the winter wheat growing season varies from 60 to 200 mm, while the average seasonal temperature varies from 10°C to 12°C. However, precipitation during the 2017–2018 and 2018–2019 growing seasons was 238 and 117 mm, respectively, while average air temperature during 2017–2018 was 9.81°C and during 2018–2019 was 9.30°C. The seasonal variations in daily maximum and minimum temperature and precipitation of both growing seasons are given in **Figure 1**.

### 2.2 Experimental set-up and crop management

The experiment had a split–split plot design for 12 treatments (three replications in each treatment) that combined two lateral spacings, irrigation scheduling, and nitrogen application practices. The lateral spacing treatments were included in main plots, while irrigation and nitrogen application were in sub-plots and sub-sub plots, respectively. Two lateral spacings were LS_40_ and LS_80_ with lateral tube spacing of 40 and 80 cm, respectively. There were two irrigation scheduling treatments including IS_20_ and IS_35_ (i.e., irrigating the crop when soil water consumption reached 20 and 35 mm, respectively). The treatments related to the nitrogen application scheme were NS_50:50_ (50% of the total as basal dose and the remaining 50% as top-dressing dose), NS_25:75_ (25% of total nitrogen as basal dose and the remaining 75% as top-dressing dose), and NS_0:100_ (all of total nitrogen as a top-dressing dose). The basal nitrogen application was broadcasted at bed preparation, while the top-dressing dose was applied by fertigation in three splits at returning green stage, jointing stage, and grain filling stage.

The Zhoumai 22 cultivar of winter wheat was sown on 15 October at 180 kg/ha seed rate and 20-cm row-to-row spacing during both growing seasons. Wang et al. (2017) conducted a literature survey on nitrogen application rate and winter wheat yield in the North China Plain. They found that the application of nitrogen at 247.6 kg/ha would be the best rate for optimum wheat yield. However, keeping in view the soil characteristics, the tested amount of nitrogen fertilizer was 270 kg/ha, which was applied in the field as per experimental treatments. The top dressing of nitrogen fertilizer was performed by fertigation as dissolving urea (46.7% N) in water up to its complete dissolution, and then dissolved urea was added to the fertilizer tank. The phosphorous application rate was 105 kg/ha as calcium superphosphate (14% P_2_O_5_), and the potassium application rate was 105 kg/ha as potassium sulfate (50% K_2_O) at basal. Experimental plots were harvested on 31 May during the 2017–2018 season and on 3 June in the 2018–2019 season.

The irrigation in the field was made through groundwater drawn by underground pipelines. The pipelines were linked to pressure-regulated pumps with pressure controllers to sustain the groundwater pressure level. Further, pipelines were interlinked to polyethylene drip irrigation tubes having a diameter of 16 mm and working operating pressure of 0.1–0.15 MPa. The emitter on drip irrigation tubes was at a 20-cm distance with a discharge rate of 2.2 L/h. In each plot, the water flow meter with valve and pressure gauge was installed to monitor the accurate supply of irrigation water.

Generally, winter wheat in NCP is irrigated on soil moisture status at returning green stage (Gao et al., 2014). By considering the local irrigation practice, the first irrigation in our experiment was carried out at the same time in all experimental treatments. However, further irrigation was performed as per the experimental design. The irrigation amount applied for IS_20_ and IS_35_ was 60 and 80 mm, respectively, during the 2017–2018 season, while the corresponding values during 2018–2019 were 100 and 140 mm, respectively. The details of the nitrogen application scheme for both seasons are given in **Table 2**.

**Table 2:** Nitrogen fertilization scheme of experimental treatments.

N application time	Fertilization rate (270 kg ha^-1^)		
	NS_50:50_	NS_25:75_	NS_0:100_
**Sowing**	135	67.5	0
**Re-greening**	45	67.5	90
**Jointing**	45	67.5	90
**Grain filling**	45	67.5	90

### 2.3 Data collection

#### 2.3.1 Wheat growth and yield parameters

After returning to the green stage of winter wheat, leaf area index (LAI) and plant height were recorded at 10–15 days of intervals. At each sampling, 10 plants were randomly collected from each plot for winter wheat growth measurement. The leaf area index was recorded as measured by Jha et al. (2019). Ten plots were harvested each time, and a ruler was used to determine the length (L) and width (W) of each leaf. The leaf area per plant (LA) was recorded as per the following equation:


(1) $$LA=\frac{\sum_{i=1}^{n}A_{i}}{n}=\frac{\sum_{i=1}^{n}\left[\sum_{j=1}^{m}(L_{j}\times W_{j})\times 0.8\right]}{n}$$

Here, *n* represents the number of plants to measure leaf area (*n* = 10), *m* represents the number of leaves in an *i*th plant, *A_i_* represents the leaf area of an *i*th plant, and *L_j_* and *W_j_* depict the length and width (both calculated in cm) of a *j*th leaf in an *i*th plant, respectively.

Further, leaf area index was measured as the ratio of leaf area to total land area.


(2) $$LAI=\frac{LA\times N}{S}$$

where *N* is the total number of plants in a row of 1 m and *S* represents the row spacing fixed to 20 cm in our experiment.

Plant height was observed from 10 plants, and here, an average of their heights was provided. During the initial growth stages, plant height was taken from the ground surface to the top, while further at heading stages, plant height was considered from the ground surface to spikelet top (without awns). Further, to determine the yield components at harvest like the number of grains per spike and spike length, 10 plants were selected in each plot. The 1-m^2^ area was harvested from each plot to assess 1,000-grain weight (g), number of spikes per unit area, number of grains per spike, aboveground biomass (t/ha), and grain yield (t/ha). The grain yield was determined at 12% of moisture content after natural sun drying.

Wheat plants having uniform growth were collected randomly to measure the plant nitrogen content. Plant parts were separated and bagged in an oven at 105°C for 0.5 h and then dried till constant weight at 80°C. Afterward, the dry weight was measured, and the total nitrogen content was determined by micro-Kjeldahl digestion, distillation, and titration methods (Huang et al., 2008).

#### 2.3.2 Measurement of soil nitrogen, crop evapotranspiration, and water use efficiency

Soil samples were taken to 1-m soil depth after 5 days of irrigation to determine soil moisture and soil nitrate nitrogen (NO_3_^−^) contents. Soil samples were excavated horizontally at 0, 10, and 20 cm to the emitter in LS_40_ while at 0, 20, and 40 cm to the emitter in LS_80_. Two equal sub-samples were derived from each main sample to determine the soil moisture and nitrate nitrogen contents. The weight of the sub-sample was measured by electronic weight balance and was subjected to an oven for drying at 105°C for 24 h. The NO_3_^−^ contents were measured by keeping the soil sample with 2 M of KCl (1:10) for 1 h. Then, the NO_3_^−^ concentration was assessed via AAR Flow Analyzer (AA3-HR; Seal Analytical Inc., Mequon, WI, USA).

Crop evapotranspiration (ETc) was calculated by soil water balance equation as followed by Chen et al. (2015):


(3) ETc=I+P+U−(R+D)±ΔS

Here, *P* and *I* represent precipitation and applied irrigation, respectively; *U* is the capillary rise of groundwater upward; *R* represents surface runoff; *D* is the downward flow; Δ*S* represents the change in soil water storage up to 100-cm soil depth. In our study, surface runoff and downward drainage were neglected owing to low irrigation amount and no heavy rainfall. Further, the upward capillary rise was also considered zero, as the water-storing capacity of the experimental site was more than what restricts upward flow.

Soil water consumption was measured by subtracting the currently measured soil moisture contents from previous moisture content as given in Equation 4.


(4) ASWC=Soil moisture after nearest irrigation−Currently measured soil moisture

WUE was calculated according to El-Rahman (2009) by dividing the grain yield by evapotranspiration.


(5) $$WUE=\frac{Y}{ETc}$$

Here, *Y* represents the grain yield.

### 2.4 Statistical analysis

The collected data were organized and analyzed in Statistics 8.1 under a split–split plot design. The drip lateral spacings data were kept in the main plot, while the irrigation scheduling treatments and nitrogen application scheme were placed in the sub-plot and sub-sub plot, respectively. To assess the impacts of irrigation scheduling, nitrogen treatments, and lateral spacings on the growth, yield, and WUE of winter wheat, an analysis of variance (ANOVA) was executed. Further, treatment means were compared by using the least significant difference (LSD) at a significance level (p = 0.05) to measure any significant differences.

## 3 Results

### 3.1 Effects of LS, IS, and NS on soil environment

#### 3.1.1 Soil moisture

**Figure 2:**
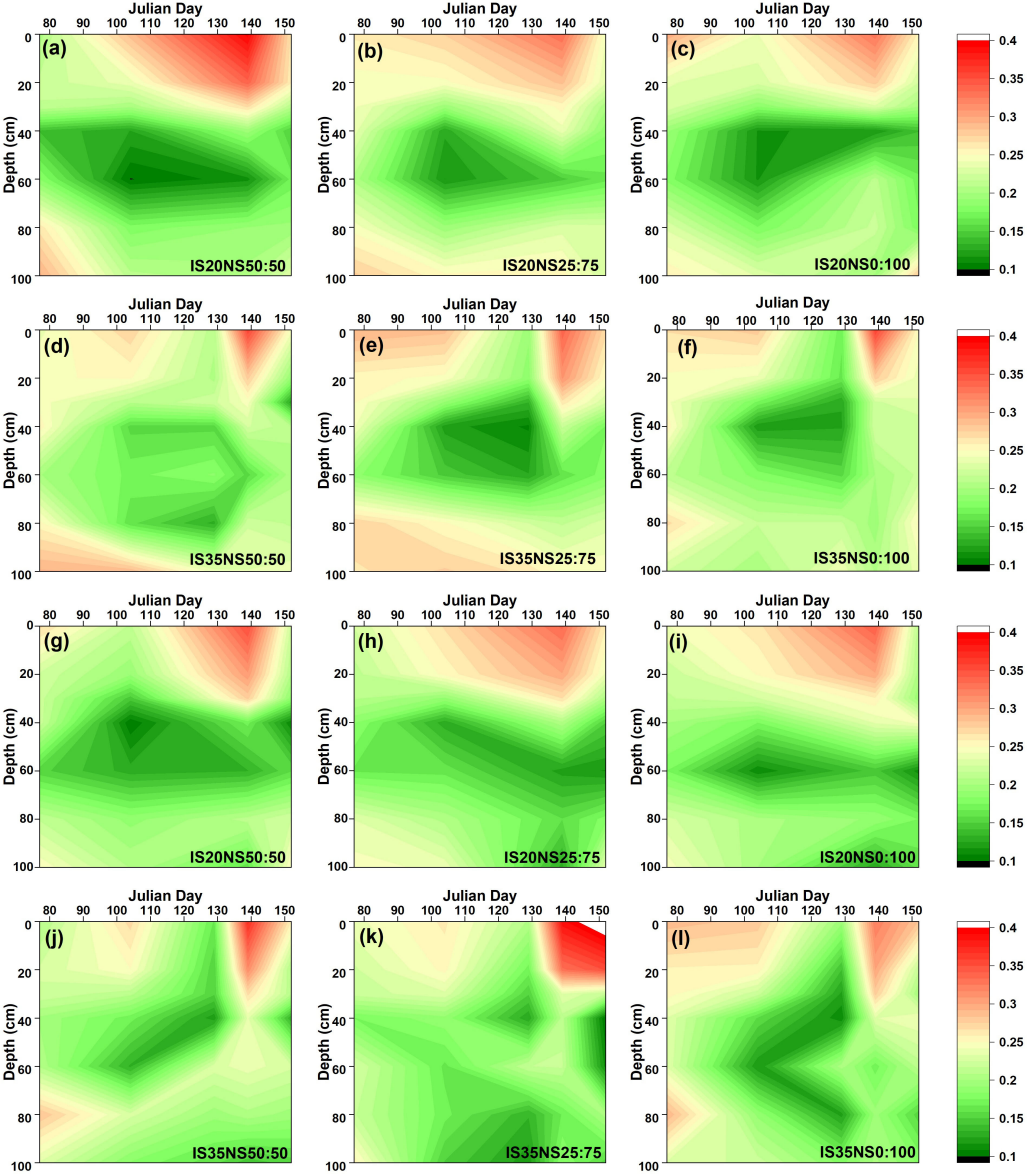
Changes in soil moisture (m^3^/m^3^) under different irrigation scheduling and nitrogen application schemes in LS_40_ **(A–F)** and LS_80_ **(G–L)** during 2017–2018 growing season.

**Figure 3:**
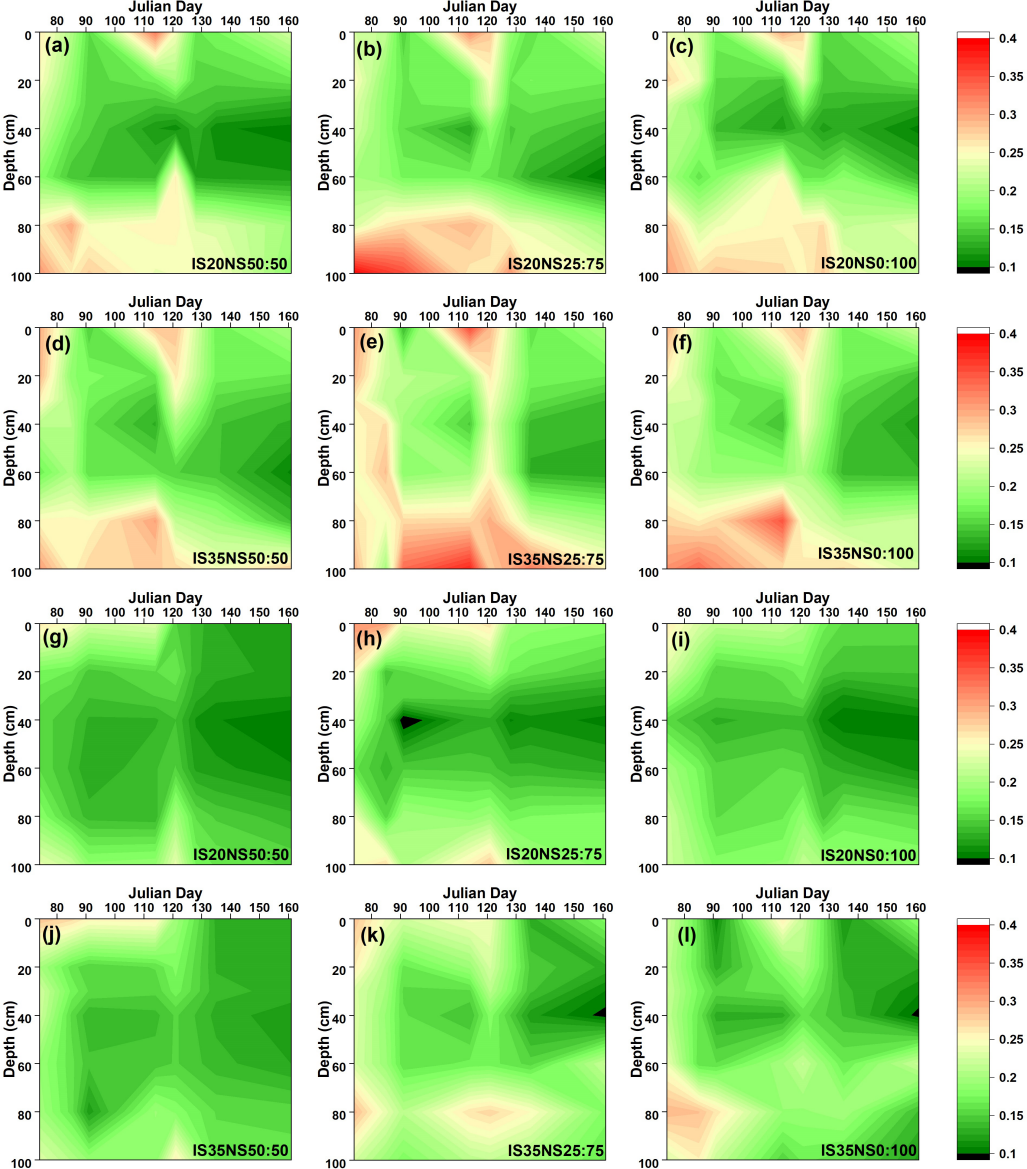
Changes in soil moisture (m^3^/m^3^) under different irrigation scheduling and nitrogen application schemes in LS_40_ **(A–F)** and LS_80_ **(G–L)** during 2018–2019 growing season.

The seasonal variations in soil moisture content during the 2017–2018 and 2018–2019 periods are illustrated in **Figures 2** and **3**, respectively. Changes in soil moisture content were notably affected due to various treatments. It was found that soil moisture contents were lower at harvest than during that the re-greening stage, as moisture contents start declining, especially after the stem-elongation stage. Due to high rainfall in the 2017–2018 growing season, soil moisture was high during the first growing season as opposed to the 2018–2019 growing season. Overall, the soil moisture was more in LS_40_ as compared to the LS_80_ during the whole growing season. Variations in soil moisture content were more prominent in upper (0–20 cm) and deeper (80–100 cm) soil depths. We observed minimum soil moisture between 20 and 60 cm and high soil moisture at the upper and deeper layers. Further, LS_40_ distributed more moisture contents in deeper soil layers than LS_80_.

#### 3.1.2 Soil nitrogen

**Figure 4:**
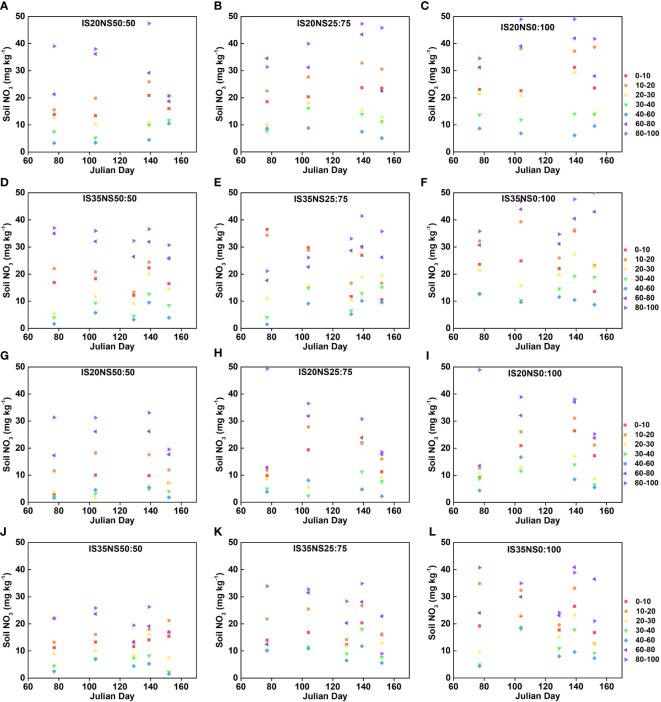
Soil nitrogen contents (mg/kg) at 100-cm depth under different irrigation scheduling and nitrogen application schemes in LS_40_ **(A–F)** and LS_80_ **(G–L)** during 2017–2018 growing season.

**Figure 5:**
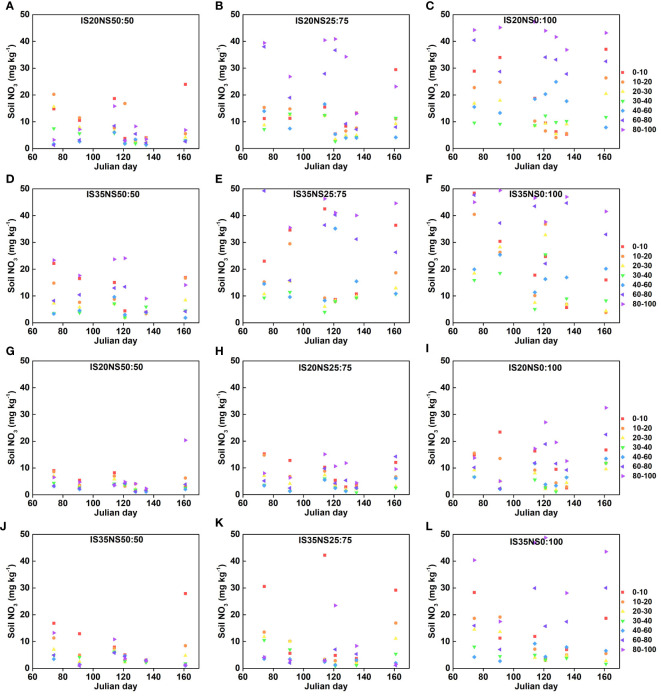
Soil nitrogen contents (mg/kg) at 100-cm depth under different irrigation scheduling and nitrogen application schemes in LS_40_ **(A–F)** and LS_80_ **(G–L)** during 2018–2019 growing season.

The seasonal changes in nitrate nitrogen (NO_3_^−^–N) contents because of different irrigation scheduling, nitrogen application scheme, and drip irrigation lateral spacing are provided in **Figures 4** and **5**, respectively. Data presented in **Figures 4**, **5** show significant differences among nitrogen application regimes. Generally, nitrate nitrogen contents increased by changing the nitrogen application from NS_50:50_ to NS_25:75_ and from NS_25:75_ to NS_0:100_. Among different lateral spacing, LS_40_ has high soil nitrogen contents during both growing seasons as compared to the LS_80_. The nitrogen application regime with more basal nitrogen application has the highest soil nitrogen at the winter wheat re-greening stage and has the lowest nitrate nitrogen at other growth stages. Overall, NO_3_ contents in IS_35_ and NS_0:100_ under 40-cm lateral spacing were the highest than that of other irrigation and nitrogen treatments. As per the experimental design, after the application of nitrogen at jointing and grain filling, soil nitrogen contents were also significantly increased. Under both irrigation treatments, soil nitrate nitrogen was mainly distributed in 0–30- and 60–100-cm soil layers. However, in IS_35_, nitrogen contents were high at a depth of 80–100 cm. Further, soil nitrogen contents observed in 40–60-cm depths were lower than those recorded in other soil depths. For most of the treatments, nitrogen contents initially showed a trend of decreasing with an increase in soil depth and then increasing in deep layers.

### 3.2 Response of crop growth indicators to different treatments

**Figure 6:**
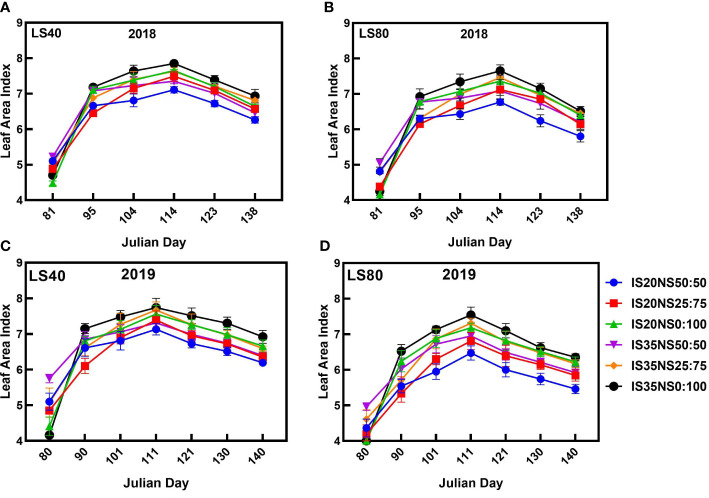
Leaf area index of winter wheat under different lateral spacings, irrigation scheduling, and nitrogen application schemes during 2018 **(A, B)** and 2019 **(C, D)** growing seasons.

**Figure 7:**
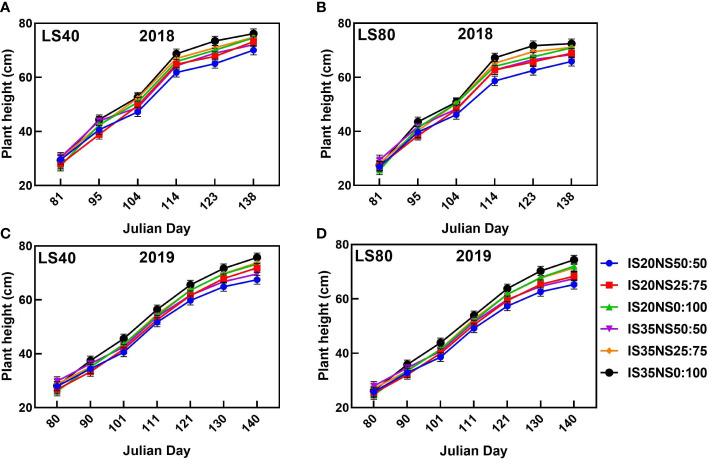
Plant height (cm) of winter wheat under different lateral spacings, irrigation scheduling, and nitrogen application schemes during 2018 **(A, B)** and 2019 **(C, D)** growing seasons.

Data presented in **Figures 6**, **7** represent the seasonal changes in winter wheat growth characteristics (leaf area index and plant height) during the 2017–2018 and 2018–2019 growing seasons under various irrigation scheduling, nitrogen application, and lateral spacing regimes. Among different treatments, leaf area index and plant height under NS_50:50_ were evidently high at the winter wheat re-greening stage. However, growth under NS_50:50_ starts decreasing and was the lowest at wheat maturity during both study seasons. The highest leaf area index and plant height were recorded in NS_0:100_ as compared to other nitrogen application scenarios. In the case of irrigation scheduling, IS_35_ increased crop growth as compared to IS_20_. Further, LS_40_ has a high plant height and leaf area index as opposed to LS_80_ during both study periods. Leaf area index values were the highest at the booting stage under all treatments. Afterward, it gradually decreased till harvesting during both growing seasons. A similar trend was noticed for growth characteristics during both study periods.

The impacts of different irrigation, nitrogen application scheme, and drip irrigation lateral spacing on the aboveground biomass of winter wheat are given in **Table 3**. Data presented in **Table 3** reveal that aboveground biomass was markedly affected due to lateral spacings (p< 0.01 and p< 0.01), irrigation scheduling (p< 0.01 and p< 0.01), and nitrogen application scheme (p< 0.001 and p< 0.001) in both growing seasons. The interaction effect of all three factors was also significant at p< 0.001 and p< 0.01 during both study seasons. We observed that increasing the irrigation amount and applying all of the total nitrogen at the re-greening, jointing, and grain-filling stages significantly enhanced winter wheat biomass in both lateral spacings. However, increasing the lateral spacing from LS_40_ to LS_80_ decreased the winter wheat aboveground biomass by 20.69% and 20.39% in both seasons, respectively. The highest wheat biomass (18.88 and 19.05 t/ha) was recorded in IS_35_NS_0:100_ under LS_40_ during the first and second growing seasons, respectively. However, the lowest aboveground biomass (8.95 and 9.26 t/ha) was noticed in IS_20_NS_50:50_ under LS_80_ during the 2017–2018 and 2018–2019 seasons, respectively.

**Table 3:** Aboveground biomass (t/ha) variability in wheat under lateral spacings, irrigation, and nitrogen application strategies.

Year	2017–2018					2018–2019			
Treatment		NS_50:50_	NS_25:75_	NS_0:100_	Average on LS	NS_50:50_	NS_25:75_	NS_0:100_	Average on LS
**LS_40_**	**IS_20_**	13.88^ef^	15.05^cd^	17.09^b^	16.26^a^	14.14^d^	15.30^c^	17.30^b^	16.48^a^
	**IS_35_**	15.27^c^	17.35^b^	18.88^a^		15.52^c^	17.57^b^	19.05^a^	
**LS_80_**	**IS_20_**	8.95^i^	12.47^h^	12.78^gh^	12.89^b^	9.26^f^	12.72^e^	12.83^e^	16.48^a^
	**IS_35_**	13.36^fg^	14.40^de^	15.39^c^		13.65^de^	14.64^cd^	15.62^c^	
**Average on NS**		12.87^c^	14.82^b^	16.04^a^		13.14^c^	15.06^b^	16.20^a^	
**Statistics analysis results**	**LS**		**			**LS**			**
	**IS**		**			**IS**			**
	**NS**		***			**NS**			***
	**LS × IS**		ns			**LS × IS**			ns
	**LS × NS**		**			**LS × NS**			*
	**IS × NS**		*			**IS × NS**			*
	**LS × IS × NS**		***			**LS × IS × NS**			**

The data are expressed as mean of three replications. Different lowercase letters represent the statistical difference at p< 0.05.

ns, non-significant difference at p< 0.05.

* Significance level at p< 0.05.

** Significance level at p< 0.01.

*** Significance level at p< 0.001.

### 3.3 Grain yield and its components

Different nitrogen application scenarios at (p< 0.001 and p< 0.001), irrigation scheduling treatments at (p< 0.001 and p< 0.001), and lateral spacings at (p< 0.05 and p< 0.01) inserted a significant effect on wheat grain yield during both years (**Table 4**). The combined interactive effect of all factors was also significant at p< 0.001 and p< 0.05 during both study periods. The grain yield values ranged from 6.89 to 8.86 t/ha during 2017–2018 and from 7.42 to 9.61 t/ha during the second study year. We found that the change in lateral spacing from LS_80_ to LS_40_ cm notably increased the grain yield by 6.95% and 7.17% in two seasons, respectively. In the case of irrigation scheduling treatments, irrigating the winter wheat at IS_35_ improved the grain yield by 15.19% and 14.18% compared with that of IS_20_ treatment during both study periods. Further, applying 25% of total nitrogen as a basal dose and the remaining 75% as a top-dressing dose evidently improved the grain yield more than other nitrogen application schemes. Overall, the highest grain yield (8.86 and 9.61 t/ha) was observed at IS_35_NS_25:75_ under 40-cm lateral spacing during both study seasons. Further, minimum winter wheat grain yield (6.89 and 7.42 t/ha) was noticed in IS_20_LS_50:50_ under LS_80_ treatment.

**Table 4:** Grain yield (t/ha) variability in wheat under lateral spacings, irrigation scheduling, and nitrogen application strategies.

Year	2017–2018					2018–2019			
Treatment		NS_50:50_	NS_25:75_	NS_0:100_	Average on LS	NS_50:50_	NS_25:75_	NS_0:100_	Average on LS
**LS_40_**	**IS_20_**	7.14^ef^	7.83^cd^	7.61^d^	8.12^a^	7.81^g^	8.49^e^	8.20^f^	8.76^a^
	**IS_35_**	8.58^ab^	8.86^a^	8.68^ab^		9.10^c^	9.61^a^	9.34^b^	
**LS_80_**	**IS_20_**	6.89^f^	7.22^e^	7.10^f^	7.59^b^	7.42^h^	7.82^g^	7.69^g^	8.17^b^
	**IS_35_**	7.85^cd^	8.40^b^	8.07^c^		8.31^ef^	9.10^c^	8.71^d^	
**Average on NS**		7.62^c^	8.08^a^	7.86^b^		8.16^c^	8.76^a^	8.49^b^	
**Statistics analysis results**	**LS**		*			**LS**			**
	**IS**		***			**IS**			***
	**NS**		***			**NS**			***
	**LS × IS**		*			**LS × IS**			ns
	**LS × NS**		ns			**LS × NS**			ns
	**IS × NS**		*			**IS × NS**			ns
	**LS × IS × NS**		***			**LS × IS × NS**			*

The data are expressed as mean of three replications. Different lowercase letters represent the statistical difference at p< 0.05.

ns, non-significant difference at p< 0.05.

* Significance level at p< 0.05.

** Significance level at p< 0.01.

*** Significance level at p< 0.001.

A mean comparison of different yield-related components including spike length, number of grains per spike, 1,000-grain weight, and spikes in a unit area under various regimes is presented in **Table 5**. Data showed that all of the yield-related indices were almost significantly changed (p< 0.01) due to lateral spacings and by p< 0.001 due to irrigation scheduling and nitrogen application scheme during both growing seasons. The combined interactive effect for spikes per unit area was non-significant in both seasons. However, the combined interactive effect for grains per spike and 1,000-grain weight was not significant during the first and second study periods, respectively. We found that a change in lateral spacings from LS_40_ cm to LS_80_ results in decreased yield components. Further, escalating the top-dressing amount of total nitrogen from 50% to 75% enhanced all yield-related parameters, but the application of all concentrations of nitrogen as a top-dressing dose reduced productivity. Application of IS_35_NS_25:75_ treatments under LS_40_ resulted in the highest spike length (8.68 cm), grains per spike (41), spikes per unit area (561 * 10^4^ ha^−1^), and 1,000-grain weight (52.94 g) during the 2017–2018 growing season; however, the corresponding values during the 2018–2019 growing season were 9.20 cm, 46.8, 711.67 * 10^4^ ha^−1^, and 53.70 g, respectively. In contrast, the lowest values for all aforementioned yield components were observed in IS_20_NS_50:50_ under 80-cm lateral spacing during both growing seasons.

**Table 5:** Interactive effects of lateral spacings, irrigation scheduling, and nitrogen application strategies on grain yield-related attributes of wheat.

Season	2017–2018				2018–2019			
Treatment	Spike length (cm)	Number of grains per spike	Spikes per unit area (10^4^ ha^−1^)	1,000-grain weight (g)	Spike length (cm)	Number of grains per spike	Spikes per unit area (10^4^ ha^−1^)	1,000-grain weight (g)
**LS_40_IS_20_NS_50:50_**	7.89^de^	28.9^f^	441.67^f^	47.57^f^	8.31^fg^	33.5^e^	511.67^i^	49.46^g^
**LS_40_IS_20_NS_25:75_**	8.38^b^	32.5^d^	483.33^d^	50.62^bcd^	8.80^bc^	37.4^c^	586.33^ef^	51.58^d^
**LS_40_IS_20_NS_0:100_**	8.05^cd^	30.2^e^	472.33^de^	50.06^cd^	8.56^de^	33.8^e^	558.33^fg^	50.89^e^
**LS_40_IS_35_NS_50:50_**	8.28^bc^	31.3^de^	487.33^cd^	50.93^bc^	8.66^cd^	35.5^d^	613.33^de^	51.55^d^
**LS_40_IS_35_NS_25:75_**	8.68^a^	41^a^	561^a^	52.94^a^	9.20^a^	46.8^a^	711.67^a^	53.70^a^
**LS_40_IS_35_NS_0:100_**	8.43^ab^	37.7^b^	513^bc^	51.58^ab^	8.98^b^	41.5^b^	673.33^b^	52.66^b^
**LS_80_IS_20_NS_50:50_**	6.71^f^	24.1^i^	398.67^g^	40.88^g^	7.26^h^	29.1^g^	475^j^	48.45^h^
**LS_80_IS_20_NS_25:75_**	8.03^cd^	25.8^gh^	451.33^ef^	48.29^ef^	8.47^ef^	30^fg^	546.67^gh^	50.16^f^
**LS_80_IS_20_NS_0:100_**	7.93^de^	25.2^hi^	431^f^	47.41^f^	8.45^ef^	29.8^fg^	521.67^hi^	49.56^g^
**LS_80_IS_35_NS_50:50_**	7.77^e^	26.6^g^	453^ef^	49.50^de^	8.16^g^	30.6^f^	588.33^e^	50.02^f^
**LS_80_IS_35_NS_25:75_**	8.33^b^	33.8^c^	521^b^	51.27^bc^	8.74^cd^	37.7^c^	645^bc^	52.19^c^
**LS_80_IS_35_NS_0:100_**	8.03^cd^	30.3^e^	473^de^	50.06^cd^	8.60^de^	34.4^e^	635^cd^	51.39^d^
**LS**	***	***	*	**	**	**	***	**
**IS**	***	***	**	***	***	***	***	***
**NS**	***	***	***	***	***	***	***	***
**LS × IS**	ns	*	ns	**	ns	*	ns	ns
**LS × NS**	***	**	ns	*	***	***	ns	ns
**IS × NS**	**	***	ns	***	**	***	ns	ns
**LS × IS × NS**	**	ns	ns	*	**	*	ns	ns

The data are expressed as mean of three replications. Different lowercase letters represent the statistical difference at p< 0.05.

ns, non-significant difference at p< 0.05.

* Significance level at p< 0.05.

** Significance level at p< 0.01.

*** Significance level at p< 0.001.

### 3.4 Changes in plant nitrogen

**Figure 8:**
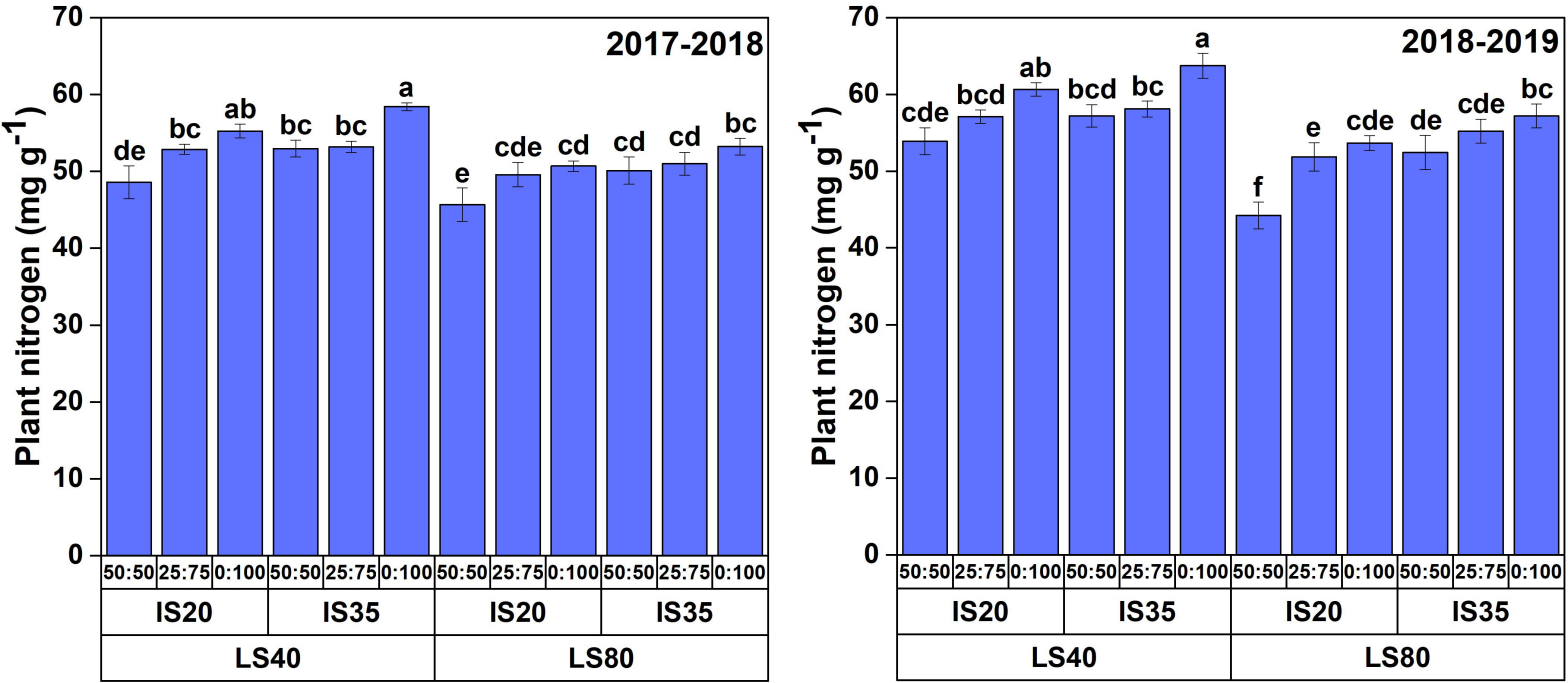
Plant nitrogen contents (mg/g) in winter wheat under various lateral spacings, irrigation scheduling, and nitrogen application regimes in 2017–2018 and 2018–2019 growing seasons. Different lowercase letters represent the statistical difference at p< 0.05.

The results regarding plant total nitrogen of both growing seasons are presented in **Figure 8**. Data revealed that irrigation scheduling, nitrogen application scheme, and lateral spacing have significantly affected the plant nitrogen content. However, their interactive effect was insignificant during both study years. In the case of drip irrigation lateral spacing, growing winter wheat at 40-cm lateral spacing improved the plant nitrogen content by 7% and 11% compared with those of LS_80_ during the first and second study periods, respectively. Between different irrigation scheduling treatments, IS_35_ improved the plant nitrogen content by 5% and 7% as opposed to IS_20_ during both study years. We found that 100% nitrogen application as a top-dressing dose enhanced the total plant nitrogen contents by 5%–10% and 6%–13% as compared to NS_25:75_ and NS_50:50_ during both seasons, respectively.

### 3.5 Effects of LS, IS, and NS on evapotranspiration and water use efficiency

The variance analysis of ETc showed that crop evapotranspiration was significantly affected due to lateral spacings (p< 0.01 and p< 0.001), irrigation scheduling (p< 0.001 in both seasons), and nitrogen application scheme (p< 0.001 in both seasons) (**Table 6**). However, their interactive effect was not significant during both study years. The average ETc values in LS_40_ and LS_80_ were 439 and 472 mm, respectively, during the first growing season, while the corresponding values in the second study period were 427 and 449 mm, respectively. In the case of irrigation scheduling treatments, irrigating the crop at IS_35_ enhanced the ETc by 11% more than that of IS_20_ in 2017–2018 and 2018–2019. The average values for ETc under IS_20_ and IS_35_ were 430 and 480 mm, respectively, during the first study period, while the corresponding values during the second study period were 414 and 462 mm, respectively. Further, increasing the top-dressing ratio also increased the ETc, as NS_0:100_ enhanced the ETc by 2% and 4% as compared to the NS_50:50_ during the first and second study years, respectively.

**Table 6:** Interactive effects of lateral spacings, irrigation scheduling, and nitrogen application strategies on ETc and WUE.

Season	2017–2018		2018–2019	
Treatment	ETc (mm)	WUE	ETc (mm)	WUE
**LS_40_IS_20_NS_50:50_**	410.48^i^	1.74^e^	398.49^i^	1.96^ef^
**LS_40_IS_20_NS_25:75_**	412^i^	1.90^ab^	403.54^hi^	2.11^ab^
**LS_40_IS_20_NS_0:100_**	419.09^h^	1.82^d^	410.31^gh^	2.00^de^
**LS_40_IS_35_NS_50:50_**	461.99^e^	1.86^bc^	441.71^e^	2.06^bc^
**LS_40_IS_35_NS_25:75_**	461.48^e^	1.92^a^	450.74^d^	2.13^a^
**LS_40_IS_35_NS_0:100_**	469.08^d^	1.85^cd^	460.24^c^	2.03^cd^
**LS_80_IS_20_NS_50:50_**	443.23^g^	1.55^h^	417.17^g^	1.78^gh^
**LS_80_IS_20_NS_25:75_**	448.48^fg^	1.61^g^	427.15^f^	1.83^g^
**LS_80_IS_20_NS_0:100_**	451.01^f^	1.57^gh^	430.21^f^	1.79^gh^
**LS_80_IS_35_NS_50:50_**	488.34^c^	1.61^g^	456.93^cd^	1.82^gh^
**LS_80_IS_35_NS_25:75_**	496.44^b^	1.70^f^	473.02^b^	1.92^f^
**LS_80_IS_35_NS_0:100_**	506.07^a^	1.60^g^	491.48^a^	1.77^h^
**LS**	**	**	***	**
**IS**	***	**	***	**
**NS**	***	***	***	***
**LS × IS**	ns	ns	ns	ns
**LS × NS**	ns	*	ns	ns
**IS × NS**	ns	**	*	ns
**LS × IS × NS**	ns	**	ns	ns

The data are expressed as mean of three replications. Different lowercase letters represent the statistical difference at p< 0.05.

ns, non-significant difference at p< 0.05; WUE, water use efficiency.

* Significance level at p< 0.05.

** Significance level at p< 0.01.

*** Significance level at p< 0.001.

Data presented in **Table 6** revealed that water use efficiency was evidently influenced by applying different irrigation scheduling (p< 0.01), nitrogen application scheme (p< 0.001), and lateral spacing (p< 0.01) treatments to winter wheat during both seasons. However, the interactive effect of all three factors was significant (p< 0.01) in the 2017–2018 growing season. An increase in WUE was observed under closer lateral spacings, as LS_40_ gave higher WUE than LS_80_. We found that LS_40_ improved the WUE by 15% and 12% as opposed to LS_80_ during both growing seasons. On average across the LS and NS, IS_35_ substantially improved the WUE by 3% and 2% as compared to IS_20_ during the 2017–2018 and 2018–2019 study periods, respectively. Concerning the NS, escalating top-dressing nitrogen from NS_50:50_ to NS_25:75_ notably improved the WUE by 5% in the first and second study seasons, respectively.

## 4 Discussion

### 4.1 Soil environment

We found that soil moisture distribution was larger in LS_40_ as compared to LS_80_. The dense lateral pipes might allow uniform moisture distribution, which results in more soil moisture content in LS_40_. These findings corroborate the findings of Chouhan et al. (2015). Different crop behaviors under various lateral spacing have also led to varied soil moisture distribution. Interestingly, we noticed that soil water contents in LS_80_ depleted slightly earlier than LS_40_, which indicates a short stress to winter wheat plants during the whole growing season.

Nitrogen applied to the agricultural system has mainly three destinations: uptake by plants, residual soil nitrogen, and loss by the soil system. Garnier et al. (2010) and Van Der Laan et al. (2010) stated that loss of nitrogen from the soil system might lead to a marked increase in groundwater nitrogen accumulation and can be a threat to aquatic life. However, the main form of nitrogen for plant uptake is NO_3_^−^–N, which can affect plant water and nutrient uptake (Nie et al., 2012). We found high nitrogen contents in deep soil layers under NS_50:50_ at the re-greening stage. This indicates that during the early development stages, wheat plants were very small and did not require much nitrogen. Thus, the application of 50% nitrogen as basal in NCP might be a loss of nitrogen resources. Zhao and Si (2015) found that nitrogen is lost through immobilization, ammonia volatilization, and denitrification during 5 months’ time span between the sowing and re-greening stages of winter wheat. The low soil nitrogen in NS_25:75_ treatment could be caused by better nitrogen utilization by wheat crops. Ma et al. (2021) stated that an adequate supply of nitrogen fertilizer guaranteed reduced nitrogen losses, rational soil distribution during crop growth, and notably improved wheat biomass in later growth stages. Further, NS_0:100_ treatment also had environmental pollution risk mainly because of surplus nitrogen in deeper soil layers at maturity. These results are similar to the findings of Shi et al. (2012) and Zain et al. (2021). As the nitrogen contents were more in deeper soil layers, we could not conclude about no leaching beyond the wheat root zone. Further, loss of nitrogen through gas emission should be recommended to address the sustainable utilization of nitrogen in winter wheat production.

### 4.2 Crop growth

Aboveground biomass is an important crop growth indicator, as it represents the inclusive contribution of plant height and leaf area index. The growth indicators of wheat including leaf area index, plant height, and aboveground biomass are presented in **Figures 6**, **7** and **Table 3**. We found that closer lateral spacings, IS_35_, and application of all the total nitrogen as the top-dressing dose guaranteed higher winter wheat growth as compared to the other treatments. Consistent with our findings, Chen et al. (2015) and Lv et al. (2019) also reported high LAI under closer drip irrigation lateral spacings. This might be probably because of water deficit conditions due to more evaporation under LS_80_ that results in reduced crop growth. We found the highest crop growth indicators under IS_35 and_ NS_0:100_, implying that sufficient irrigation and nitrogen were present in the field for winter wheat growth. Abrar et al. (2020) and Si et al. (2020) found that nitrogen availability positively affects crop growth, while water deficiency negatively influenced crop growth. Deficient irrigation and fertilizer lead to a reduction in different cell processes, like cell division and cell elongation (Farooq et al., 2009), thereby reducing the LAI, plant height, and aboveground biomass under IS_20_ and NS_50:50_ treatments. Xu et al. (2021) also reported improved crop growth with a top-dressing dose of nitrogen fertilizer under a wheat–maize intercropping system. High leaf area index values and plant height under NS_50:50_ at the re-greening stage and then lower corresponding values at the next growth stages indicate that applying all of the total nitrogen as top-dressing dose could be just beneficial for an increase in winter wheat growth indicators.

### 4.3 Plant N uptake

An adequate nitrogen supply is critical for the normal growth and development of crops. Srivastava et al. (2018) reported that the fertilizer amount available for plant uptake directly influences crop growth and development, thereby affecting the final crop yield. We found that increasing the top-dressing ratio led to higher nitrogen accumulation in wheat plants. Previously, Rasmussen et al. (2015); Liu et al. (2017), and Yin et al. (2020) reported that an increase in nitrogen application results in high nitrogen uptake. Zhang et al. (2020) also stated that plant nitrogen concentration was significantly increased by top-dressing nitrogen under various irrigation treatments. In the current study, IS_35_ and NS_0:100_ significantly enhanced the nitrogen uptake by wheat plants. These results are similar to those of Guo et al. (2015), who reported higher nitrogen uptake in maize plants with an increase in irrigation and nitrogen supply. To the best of our knowledge, this is the first study in which nitrogen uptake by wheat plants was analyzed under different drip irrigation lateral spacings. We found higher nitrogen uptake by plants under LS_40_, which might be caused by the optimum availability of water content as compared to the water availability in LS_80_. Conclusively, considering the wheat yield and WUE, applying 25% of total nitrogen as basal and the remaining 75% as top-dressing dose can be a suitable scheme for sustainable winter wheat productivity.

### 4.4 Grain yield and its components

Elucidating the most efficient lateral spacing, irrigation scheduling practice, and nitrogen application scheme was the key concern of this study. The ultimate objective of the study was to improve the grain yield for better sustainable agriculture. We found that grain yield was highly variable across different experimental treatments (**Table 4**). The rise in grain yield under LS_40_ might be attributed to high values of grain yield components under closer lateral spacings. Xu et al. (2018) stated that grain yield is closely associated with spikelets per spike and the number of grains per spike. In our study, yield components, especially the number of grains per spike and 1,000-grain weight, showed poor performance under LS_80_. These results are similar to the findings of Lv et al. (2019), who found 9.96% and 8.52% reduction in grain yield of widened lateral spacings compared with closer lateral tubes. The reduced number of grains per spike and 1,000-grain weight were the major drivers for this decrease in yield as presented in **Table 5**. Irrigation scheduling has also an important role in winter wheat productivity, as the highest yield was obtained under IS_35_-related treatments. Mostafa et al. (2018) found that continuous availability of moisture in wheat root zone led to highest grain yield and yield-related parameters while applying double lateral lines than that of single drip lateral line.

The presence of enough nitrogen is also very important to achieve better winter wheat production. The continuous decreasing trend in NS_50:50_ was noticed in the current study, as the leaf area index, plant height, aboveground biomass, and grain yield components were all lower in NS_50:50_ as compared to the other nitrogen application scheme. We found the best winter wheat grain yield by applying 75% nitrogen as top-dressing dose rather than 50% at top-dressing dose. These results are in line with the findings of Liu et al. (2019), who stated that decreasing basal application and increasing top-dressing dose remarkably enhance the yield components and finally the yield of winter wheat. Moreover, growth indicators were high in NS_0:100_, but the grain yield and yield-related attributes were not better in this application scheme. More application of nitrogen as top-dressing dose might delay the wheat maturity and enhance the growth characteristics rather than yield parameters. Thus, the top-dressing dose of all nitrogen is not a good application strategy for winter wheat. Liang et al. (2017) and Zhang et al. (2017) stated that the application of more nitrogen at the booting stage might influence the grain filling and ultimately the grain yield by delaying maturity.

### 4.5 ETc and WUE

The ETc values were high in the 2017–2018 growing season as compared to the 2018–2019 season. This could be mainly because of more precipitation (approximately 238 mm) in the first study season than 117-mm rainfall in the second growing season. A significant difference in ETc between LS_40_ and LS_80_ was observed during both growing seasons. The higher ETc values in LS_80_ might be due to the more evaporation losses, as the distance among drip laterals was so large to not facilitate the proper moisture content distribution for wheat growth and productivity, while less ETc values under 40-cm drip lateral spacing may relate to proper moisture availability for wheat plants. In the case of the nitrogen application scheme, the application of all nitrogen as top-dressing dose notably enhanced the ETc during both growing seasons. This is likely a result of high plant growth under this treatment, as high plant growth requires much water from the soil and also results in more transpiration. The highest plant growth under NS_0:100_ could be the reason for more ETc in this treatment (Qi et al., 2009). Further, our results are in line with the findings of Rathore et al. (2017), who found 8% enhanced ETc in plants treated with more nitrogen as compared to plants treated with low nitrogen.

In terms of low grain yield and high ETc under LS_80_, WUE was also low under this drip irrigation lateral spacing. However, the higher WUE under LS_40_ could be mainly because of more grain yield and less ETc. Mostafa et al. (2018) found that growing the plants in double drip lateral lines notably enhanced the WUE more than that of single lateral lines. Among different nitrogen application treatments, increasing the top-dressing ratio from 50% to 75% significantly improved the WUE, but a further increase in nitrogen application from 75% to 100% as top-dressing dose decreased the WUE. This is likely caused by more grain yield under NS_25:75_ and less ETc than NS_0:100_ treatment. Further, these findings are in parallel with the results of El-Hendawy et al. (2008) and Zhang et al. (2017). We suggest that proper splitting of total nitrogen fertilizer is advantageous to improve the WUE rather than increase in application rate.

## 5 Conclusion

Soil water and nitrogen resources are suffering from overuse; alarmingly, this situation is projected to worsen in the near future. Therefore, management of these resources by doing field studies is necessary for sustainable agricultural productivity. In our study, we highlighted that different drip lateral spacings, irrigation scheduling, and nitrogen fertilization scheme applied over two consecutive growing seasons have a clear influence on winter wheat growth and productivity. The irrigation scheduling whenever soil water consumption reached 35 mm plus 25% nitrogen as the basal dose and 75% as the top-dressing dose under drip lateral spacing of 40 cm evidently facilitated the winter wheat yield, yield-related parameters, and water use efficiency. In contrast, growing winter wheat under 80-cm lateral spacing with other irrigation scheduling and nitrogen fertilization treatments was not a suitable choice for winter wheat productivity. Further, in order to prevent nitrogen loss in winter wheat, the treatment with NS_0:100_ is not recommended, as this treatment just improved the growth characteristics of winter wheat and did not produce suitable grain yield. Further, studies on the physiological and biochemical processes of winter wheat under different irrigation scheduling and nitrogen fertilization management strategies should be considered for a better understanding of winter wheat growth and yield pattern under varied soil and environmental conditions. We also suggest that nitrogen loss through greenhouse gas emission should also be quantified by conducting experiments under various split nitrogen application regimes. Finally, genetic expression must be taken into consideration to better understand the underlying mechanisms under different treatments.

## Data availability statement

The original contributions presented in the study are included in the article/supplementary material. Further inquiries can be directed to the corresponding authors.

## Author contributions

Investigation: MZ. Methodology: MZ and FM. Data curation: MZ, CM, and AK. Formal analysis: MZ, ZS, HM, FM, and CS. Writing—original draft: MZ and ZS. Visualization: ZS. Software: HM, CM, and AK. Supervision: DA and CS. Writing—review and editing: DA and CS. All authors contributed to the article and approved the submitted version.
